# Suicide attempts among activated soldiers in the U.S. Army reserve components

**DOI:** 10.1186/s12888-018-1978-2

**Published:** 2019-01-18

**Authors:** James A. Naifeh, Robert J. Ursano, Ronald C. Kessler, Oscar I. Gonzalez, Carol S. Fullerton, Holly B. Herberman Mash, Charlotte A. Riggs-Donovan, Tsz Hin Hinz Ng, Gary H. Wynn, Hieu M. Dinh, Tzu-Cheg Kao, Nancy A. Sampson, Murray B. Stein

**Affiliations:** 1 Center for the Study of Traumatic Stress, Department of Psychiatry, Uniformed Services University of the Health Sciences, 4301 Jones Bridge Road, Bethesda, MD 20814 USA; 2 Department of Health Care Policy, Harvard Medical School, 180 Longwood Avenue, Boston, MA 02115 USA; 3 Department of Preventive Medicine and Biostatistics, Uniformed Services University of the Health Sciences, 4301 Jones Bridge Road, Bethesda, MD 20814 USA; 4 Departments of Psychiatry and Family Medicine & Public Health, University of California San Diego, 8939 Villa La Jolla Drive, Suite 200, La Jolla, CA 92037 USA; 5 VA San Diego Healthcare System, 8810 Rio San Diego Drive, San Diego, CA 92108 USA

**Keywords:** Military, Suicide attempt, Risk factors, Army National Guard, Army reserve

## Abstract

**Background:**

Although the majority of active duty U.S. Army soldiers are full-time personnel in the Active Component (AC), a substantial minority of soldiers on active duty are in the Reserve Components (RCs). These “citizen-soldiers” (Army National Guard and Army Reserve) represent a force available for rapid activation in times of national need. RC soldiers experience many of the same stressors as AC soldiers as well as stressors that are unique to their intermittent service. Despite the important role of RC soldiers, the vast majority of military mental health research focuses on AC soldiers. One important goal of the Army Study to Assess Risk and Resilience in Servicemembers (Army STARRS) is to address this gap. Here we examine predictors of suicide attempts among activated RC soldiers.

**Methods:**

This longitudinal, retrospective cohort study used individual-level person-month records from Army and Department of Defense administrative data systems to examine socio-demographic, service-related, and mental health predictors of medically documented suicide attempts among activated RC soldiers during 2004–2009. Data from all 1103 activated RC suicide attempters and an equal-probability sample of 69,867 control person-months were analyzed using a discrete-time survival framework.

**Results:**

Enlisted soldiers comprised 84.3% of activated RC soldiers and accounted for 95.7% of all activated RC suicide attempts (overall rate = 108/100,000 person-years, more than four times the rate among officers). Multivariable predictors of enlisted RC suicide attempts included being female, entering Army service at age ≥ 25, current age < 30, non-Hispanic white, less than high school education, currently married, having 1–2 years of service, being previously deployed (vs. currently deployed), and history of mental health diagnosis (particularly when documented in the previous month). Predictors among RC officers (overall rate = 26/100,000 person-years) included being female and receiving a mental health diagnosis in the previous month. Discrete-time hazard models showed suicide attempt risk among enlisted soldiers was inversely associated with time in service.

**Conclusions:**

Risk factors for suicide attempt in the RCs were similar to those previously observed in the AC, highlighting the importance of research and prevention focused on RC enlisted soldiers in the early phases of Army service and those with a recent mental health diagnosis.

**Electronic supplementary material:**

The online version of this article (10.1186/s12888-018-1978-2) contains supplementary material, which is available to authorized users.

## Background

Rates of non-fatal suicide attempts and suicide deaths among U.S. Army soldiers increased during the wars in Iraq and Afghanistan [1–3], with elevated risk persisting among veterans who have left the military [4, 5]. Most research on non-fatal suicide attempts has focused on the full-time soldiers within the Army’s Active Component (AC) (e.g., [6–8]). Suicide attempts among soldiers in the Reserve Components (RCs), which also increased during the wars [9], have received much less attention. The RCs include the Army National Guard and Army Reserve, comprising approximately 53% of the total Army population (all active and inactive soldiers) in 2017 [10]. These “citizen-soldiers” augment the AC as needed during wartime, returning to their communities, civilian jobs, and/or college studies after deployment. In addition to their unique role and circumstances, RC soldiers may differ from the AC on mental health outcomes (e.g., stress, anxiety, and depression) and risk for suicide ideation and attempt [11–14]. Improved understanding of suicide attempt risk within the RCs can inform targeted intervention programs for this substantial proportion of the Army population.

Most previous findings on non-fatal suicidal events within the RCs are based on survey research (e.g., [15–17]). Few studies have examined suicide attempts documented in the administrative medical records of RC soldiers, which are particularly important owing to their impact on the Army healthcare system. Univariable analyses indicate that documented attempts are more likely among RC soldiers who are female, younger, less educated, and never married [9]. Here we used 2004–2009 administrative data from the Army Study to Assess Risk and Resilience in Servicemembers (Army STARRS) to examine multivariable associations of socio-demographic, service-related, and mental health predictors with suicide attempts among activated RC soldiers.

## Methods

### Sample

The Historical Administrative Data Study (HADS) is a component of Army STARRS that integrates deidentified records from 38 Army and Department of Defense administrative data systems [18]. The study was approved by the institutional review boards of the Uniformed Services University, University of California, San Diego, Harvard Medical School, and University of Michigan Institute for Social Research. In this longitudinal, retrospective cohort study, we focused on the active-duty person-month records for the 743,171 RC soldiers (i.e., U.S. Army National Guard and U.S. Army Reserve) who were federally activated under Title 10 from January 1, 2006 through December 31, 2009. During that time there were 1103 RC soldiers with a documented suicide attempt (1056 enlisted soldiers, 47 commissioned and warrant officers). Person-month data were analyzed using a discrete-time survival framework [19], with each month in a soldier’s career treated as a separate observational record. In order to reduce computational intensity, we selected an equal-probability 1:200 sample of 69,867 control person-months (58,895 enlisted soldiers, 10,972 officers). When control person-months are randomly subsampled and weighted, discrete-time survival coefficients can be estimated without bias [20]. Prior to selection, the population of control person-months was stratified by gender, rank, time in service, deployment status (never deployed, currently deployed, or previously deployed), and historical time. The control sample excluded all person-months in which a soldier died or had an administratively documented non-fatal suicidal event (e.g., suicide attempt, suicide ideation) [3]. To adjust for under-sampling, control person-months were weighted to 200.

### Measures

Administratively documented suicide attempts were identified using Department of Defense Suicide Event Report (DoDSER) records and ICD-9-CM E950-E958 diagnostic codes (indicating self-inflicted poisoning or injury with suicidal intent). The DoDSER is a suicide surveillance program in which medical providers at DoD treatment facilities use a standardized reporting form to document suicidal behaviors [21]. The Military Health System Data Repository, Theater Medical Data Store, and TRANSCOM (Transportation Command) Regulating and Command and Control Evacuating System (Additional file 1: Table S1) were used to identify ICD-9-CM E950-E958 codes documented during healthcare encounters at military and civilian treatment facilities, as well as during combat operations and aeromedical evacuations. Records indicating suicide death or only suicide ideation (without an attempt) were excluded. We also excluded the E959 code (late effects of a self-inflicted injury) because it confounds the temporal associations between risk factors and suicide attempts [22]. Cross-referencing of the different data systems was conducted to ensure that all cases represented unique soldiers. When multiple suicide attempts were documented for a single soldier, a hierarchical classification scheme was used to select the first attempt [3]. Administrative records were also used to construct variables for socio-demographic characteristics (gender, age at entry into Army service, current age, race, education, and marital status), active time in service (based on the number of months an RC soldier was activated), deployment status (never deployed, currently deployed, or previously deployed), and previous mental health diagnosis (Additional file 1: Table S1). The indicator for any previous mental health diagnosis was constructed using ICD-9-CM mental disorder codes, excluding postconcussion syndrome, tobacco use disorder, and supplemental V-codes (Additional file 1: Table S2). Recency of mental health diagnosis was determined by calculating the number of months between the most recently recorded diagnosis and the subsequent suicide attempt (cases) or sampled person-month (controls).

### Analysis methods

Analyses were conducted using SAS version 9.4 [23]. Enlisted soldiers and officers were examined separately owing to their different socio-demographic profiles, Army training and career experiences [24], and risk of mental health problems [25–27] and suicidal behaviors [8, 28–30]. Discrete-time person-month survival analysis with a logistic link function was used to examine multivariable associations of socio-demographic characteristics with suicide attempt. This was followed by a series of multivariable models (adjusting for the socio-demographic variables) that separately examined the incremental predictive effects of active time in service, deployment status, and presence/recency of a previous mental health diagnosis. Due to the small number of activated RC officers with a documented suicide attempt, some of the predictor categories had to be combined in subsequent officer analyses. Odds-ratios (ORs) and 95% confidence intervals (CI) were obtained by exponentiating the logistic regression coefficients. Using coefficients from the final model, we generated a *standardized* risk estimate (SRE; [31]) (number of suicide attempters per 100,000 person-years) for each predictor category. The SREs assume other predictors in the final model are at their sample-wide means. A dummy predictor for calendar month and year was included in all logistic regression equations to control for secular trends in suicidal behaviors during the study period [1–3, 9, 32]. Among enlisted soldiers (the number of officer cases was insufficient), changes in suicide attempt risk across active time in service were further examined in a discrete-time survival model that estimated risk (suicide attempters per 100,000 person-months) in each active month since entering the Army.

## Results

### RC suicide attempt rates by rank

Enlisted soldiers comprised 84.3% of activated RC soldiers, accounted for 95.7% of activated RC suicide attempters (*n* = 1056), and had an overall suicide attempt rate of 108/100,000 person-years (95% CI = 106.8–108.3). Officers (commissioned and warrant officers) made up 15.7% of activated RC soldiers and accounted for 4.3% of attempters (*n* = 47), with an overall rate of 26/100,000 person-years (95% CI = 25.3–26.1). The overall suicide attempt rate for activated RC enlisted soldiers was more than four times higher than the rate for officers (rate ratio = 4.2 [95% CI = 3.1–5.6]).

### Socio-demographic characteristics

Among enlisted RC soldiers, females were more than twice as likely as males to have a documented suicide attempt. Those younger than 21 years had the highest odds of suicide attempt (OR = 3.9 [95% CI = 3.0–5.1]), with odds decreasing monotonically as age increased. Odds were also elevated among those who were less than high school-educated (OR = 1.2 [95% CI = 1.1–1.4]), currently married (OR = 1.5 [95% CI = 1.3–1.8]), and 25 years or older when they entered the Army (OR = 1.3 [95% CI = 1.0–1.6]). Odds of suicide attempt were lower among soldiers who were Black or Asian (ORs = 0.5–0.8), 40 years or older (OR = 0.6 [95% CI: 0.5–0.8]), college-educated (OR = 0.6 [95% CI = 0.4–0.8]), and younger than 21 when they entered the Army (OR = 0.6 [95% CI = 0.5–0.7]). Standardized risk of suicide attempt was highest for enlisted RC soldiers who were younger than 21 (SRE = 293/100,000 person-years), female (SRE = 221/100,000 person-years), and 25 years or older when they entered the Army (SRE = 203/100,000 person-years) (Table 1). The only socio-demographic variable associated with suicide attempt among officers was gender, with odds being more than three times higher among females (OR = 3.3 [95% CI = 1.7–6.1]) (Table 2). Female gender was also the socio-demographic category with highest standardized risk in the officer population (SRE = 61/100,000 person-years).

**Table 1:** Multivariable associations of socio-demographic characteristics with suicide attempt among activated enlisted soldiers in the U.S. Army reserve components^a^

	OR	(95% CI)	Cases (*N*)	Total (*N*)^b^	Rate^c^	Pop %^d^	SRE^e^
Gender							
Male	1.0	–	713	9,951,713	86	84.5	86
Female	2.6*	(2.2–2.9)	343	1,828,343	225	15.5	221
χ^2^_1_	188.4*						
Age at Army Entry (years)							
< 21	0.6*	(0.5–0.7)	748	8,657,748	104	73.5	93
21–24	1.0	–	189	1,937,989	117	16.4	157
≥ 25	1.3*	(1.0–1.6)	119	1,184,319	121	10.1	203
χ^2^_2_	60.7*						
Current Age (years)							
< 21	3.9*	(3.0–5.1)	354	1,805,754	235	15.3	293
21–24	1.9*	(1.4–2.4)	225	2,180,025	124	18.5	137
25–29	1.4*	(1.1–1.8)	184	1,969,584	112	16.7	105
30–34	1.0	–	101	1,458,301	83	12.4	73
35–39	0.8	(0.6–1.1)	85	1,622,885	63	13.8	59
≥ 40	0.6*	(0.5–0.8)	107	2,743,507	47	23.3	45
χ^2^_5_	220.1*						
Race/Ethnicity							
White	1.0	–	752	7,954,552	113	67.5	116
Black	0.8*	(0.7–1.0)	163	2,065,363	95	17.5	93
Hispanic	0.8	(0.7–1.0)	98	1,144,298	103	9.7	97
Asian	0.5*	(0.3–0.7)	19	403,819	57	3.4	53
Other	1.2	(0.8–1.8)	24	212,024	136	1.8	136
χ^2^_4_	18.8*						
Education							
< High School^f^	1.2*	(1.1–1.4)	276	1,832,476	181	15.6	132
High School	1.0	–	688	7,961,288	104	67.6	106
Some College	0.9	(0.6–1.2)	42	706,842	71	6.0	93
≥ College	0.6*	(0.4–0.8)	50	1,279,450	47	10.9	63
χ^2^_3_	20.3*						
Marital Status							
Never Married	1.0	–	620	5,846,020	127	49.6	92
Currently Married	1.5*	(1.3–1.8)	397	5,392,597	88	45.8	142
Previously Married	1.4	(1.0–2.0)	39	541,439	86	4.6	132
χ^2^_2_	29.8*						
Total	–	–	1056	11,780,056	108	100.0	–

^a^The sample of activated Reserve Component enlisted soldiers (n = 1056 cases, 58,895 control person-months) is a subset of the total Reserve Component sample (*n* = 70,970 person-months) from the Army STARRS Historical Administrative Data Study (HADS). All control person-months were assigned a weight of 200 to adjust for under-sampling. The analysis included a dummy predictor variable for calendar month and year to control for secular trends

^b^Total includes both cases (i.e., suicide attempters) and weighted control person-months

^c^Rate per 100,000 person-years, calculated based on n_1_/n_2_, where n_1_ is the unique number of soldiers within each category and n_2_ is the annual number of person-*years*, not person-*months*, in the population

^d^*Pop %* Percentage of the activated Reserve Component enlisted population

^e^*SRE* Standardized risk estimate (suicide attempters per 100,000 person-years) was calculated assuming other predictors were at their sample-wide means

^f^< High School includes: General Educational Development credential (GED), home study diploma, occupational program certificate, correspondence school diploma, high school certificate of attendance, adult education diploma, and other non-traditional high school credentials

*p < 0.05

**Table 2:** Multivariable associations of socio-demographic characteristics with suicide attempt among activated officers in the U.S. Army reserve components^a^

	OR	(95% CI)	Cases (*N*)	Total (*N*)^b^	Rate^c^	Pop %^d^	SRE^e^
Gender							
Male	1.0	–	30	1,858,430	19	84.7	19
Female	3.3*	(1.7–6.1)	17	336,017	61	15.3	61
χ^2^_1_	13.7*						
Age at Army Entry (years)							
< 21	0.9	(0.5–1.8)	22	1,158,822	23	52.8	24
21–24	1.0	–	15	694,415	26	31.6	25
≥ 25	1.3	(0.6–3.0)	10	341,210	35	15.5	34
χ^2^_2_	0.8						
Current Age (years)							
≤ 29	0.7	(0.3–2.0)	7	231,407	36	10.5	38
30–34	1.0	–	10	280,610	43	12.8	46
35–39	0.4	(0.2–1.1)	8	486,008	20	22.1	21
≥ 40	0.4*	(0.2–0.9)	22	1,196,422	22	54.5	21
χ^2^_3_	5.7						
Race/Ethnicity							
White	1.0	–	38	1,688,438	27	76.9	29
Non-White	0.6	(0.3–1.3)	9	506,009	21	23.1	18
χ^2^_1_	1.5						
Education							
≤ College	1.0	–	31	1,511,631	25	68.9	24
> College	1.2	(0.7–2.4)	16	682,816	28	31.1	30
χ^2^_1_	0.5						
Marital Status							
Never/Previously Married	1.0	–	19	676,219	34	30.8	28
Currently Married	0.9	(0.5–1.6)	28	1,518,228	22	69.2	24
χ^2^_1_	0.2						
Total			47	2,194,447	26	100.0	–

^a^The sample of activated Reserve Component officers (n = 47 cases, 10,972 control person-months) is a subset of the total Reserve Component sample (*n* = 70,970 person-months) from the Army STARRS Historical Administrative Data Study (HADS). All control person-months were assigned a weight of 200 to adjust for under-sampling. The analysis included a dummy predictor variable for calendar month and year to control for secular trends

^b^Total includes both cases (i.e., suicide attempters) and weighted control person-months

^c^Rate per 100,000 person-years, calculated based on n_1_/n_2_, where n_1_ is the unique number of soldiers within each category and n_2_ is the annual number of person-*years*, not person-*months*, in the population

^d^*Pop %* Percentage of the activated Reserve Component officer population

^e^SRE Standardized risk estimate (suicide attempters per 100,000 person-years) was calculated assuming other predictors were at their sample-wide means

*p < 0.05

### Active time in service

Adjusting for socio-demographic variables, enlisted RC soldiers in their first two active years of service had higher odds of suicide attempt relative to those with 5–10 active years of service (OR = 1.9 [95% CI = 1.4–2.5]), whereas those with more than 10 active years of service had lower odds (OR = 0.7 [95% CI = 0.5–1.0]) (Table 3). Subsequent pairwise analysis found that the ORs for 1–2 years (0–24 months) and 3–4 years (25–48 months) of service also differed (χ^2^_1_ = 17.5, *p* < 0.0001). The standardized risk for enlisted RC personnel with 1–2 years of service (SRE = 160/100,000 person-years) was nearly three times higher than for those with more than 10 years of service (SRE = 55/100,000 person-years) (Table 3). Time in service was not associated with suicide attempt among officers (Table 4).

**Table 3:** Multivariable associations of active time in service, deployment status, and time since most recent mental health diagnosis with suicide attempt among activated enlisted soldiers in the U.S. Army reserve components^a,b^

	OR	(95% CI)	Cases (*N*)	Total (*N*)^c^	Rate^d^	Pop %^e^	SRE^f^
I. Active Time in Service^b,^^g^							
1–2 years	1.9*	(1.4–2.5)	651	4,043,051	193	34.3	160
3–4 years	1.1	(0.9–1.5)	111	1,441,711	92	12.2	96
5–10 years	1.0	–	126	1,873,726	81	15.9	84
> 10 years	0.7*	(0.5–1.0)	168	4,421,568	46	37.5	55
χ^2^_3_	32.0*						
II. Deployment Status^b^							
Never deployed	1.2	(1.0–1.4)	634	5,864,234	130	49.8	108
Currently deployed	1.0	–	230	3,393,630	81	28.8	93
Previously deployed	1.3*	(1.1–1.6)	192	2,522,192	91	21.4	129
χ^2^_2_	7.4*						
III. Time Since Most Recent Mental Health Diagnosis^b^							
No Diagnosis	1.0	–	580	10,198,180	68	86.6	65
1 Month	28.4*	(24.7–32.7)	330	254,530	1556	2.2	1863
2–3 Months	6.0*	(4.6–7.8)	61	217,661	336	1.8	399
4–12 Months	2.8*	(2.1–3.7)	52	438,652	142	3.7	184
≥ 13 Months	1.5*	(1.0–2.1)	33	671,033	59	5.7	101
χ^2^_4_	2215.1*						

^a^The sample of activated Reserve Component enlisted soldiers (*n* = 1056 cases, 58,895 control person-months) is a subset of the total Reserve Component sample (*n* = 70,970 person-months) from the Army STARRS Historical Administrative Data Study (HADS). All control person-months were assigned a weight of 200 to adjust for under-sampling

^b^Time in service, deployment status, and mental health diagnosis were each examined in a separate model that controlled for basic socio-demographic variables (gender, age at entry into the Army, current age, race, education, marital status). All analyses also included a dummy predictor variable for calendar month and year to control for secular trends

^c^Total includes both cases (i.e., suicide attempters) and weighted control person-months

^d^Rate per 100,000 person-years, calculated based on n_1_/n_2_, where n_1_ is the unique number of soldiers within each category and n_2_ is the annual number of person-*years*, not person-*months*, in the population

^e^*Pop %* Percentage of the activated Reserve Component enlisted population

^f^*SRE* Standardized risk estimate (suicide attempters per 100,000 person-years) was calculated assuming other predictors were at their sample-wide means

^g^Based on the number of months a soldier served on active duty (e.g., 1–2 years = 0–24 months of active service; 3–4 years = 25–48 months of active service, etc.)

*p < 0.05

**Table 4:** Multivariable associations of active time in service, deployment status, and time since most recent mental health diagnosis with suicide attempt among activated officers in the U.S. Army reserve components^a,b^

	OR	(95% CI)	Cases (*N*)	Total (*N*)^c^	Rate^d^	Pop %^e^	SRE^f^
I. Active Time in Service^b,g^							
1–4 years	1.1	(0.4–3.1)	9	255,609	42	11.6	34
5–10 years	1.0	–	10	330,010	36	15.0	28
> 10 years	1.0	(0.4–2.6)	28	1,608,828	21	73.3	23
χ^2^_2_	0.1						
II. Deployment Status^b^							
Never deployed	0.9	(0.4–1.8)	23	1,014,623	27	46.2	29
Currently deployed	1.0	–	12	517,412	28	23.6	25
Previously deployed	0.7	(0.3–1.6)	12	662,412	22	30.2	24
χ^2^_2_	0.7						
III. Time Since Most Recent Mental Health Diagnosis^b^							
No Diagnosis	1.0	–	17	1,881,817	11	85.8	11
1 Month	49.6*	(24.3–100.9)	17	39,817	512	1.8	540
2–3 Months	16.4*	(6.2–43.3)	6	37,206	194	1.7	189
≥ 4 Months	3.1*	(1.3–7.7)	7	235,607	36	10.7	38
χ^2^_3_	124.3*						

^a^The sample of activated Reserve Component officers (*n* = 47 cases, 10,972 control person-months) is a subset of the total Reserve Component sample (*n* = 70,970 person-months) from the Army STARRS Historical Administrative Data Study (HADS). All control person-months were assigned a weight of 200 to adjust for under-sampling

^b^Time in service, deployment status, and mental health diagnosis were each examined in a separate model that controlled for basic socio-demographic variables (gender, age at entry into the Army, current age, race, education, marital status). All analyses also included a dummy predictor variable for calendar month and year to control for secular trends

^c^Total includes both cases (i.e., suicide attempters) and weighted control person-months

^d^Rate per 100,000 person-years, calculated based on n_1_/n_2_, where n_1_ is the unique number of soldiers within each category and n_2_ is the annual number of person-*years*, not person-*months*, in the population

^e^*Pop %* Percentage of the activated Reserve Component officer population

^f^*SRE* Standardized risk estimate (suicide attempters per 100,000 person-years) was calculated assuming other predictors were at their sample-wide means

^g^Based on the number of months a soldier served on active duty (e.g., 1–4 years = 0–48 months of active service; 5–10 years = 60–120 months of active service, etc.)

*p < 0.05

**Fig. 1:**
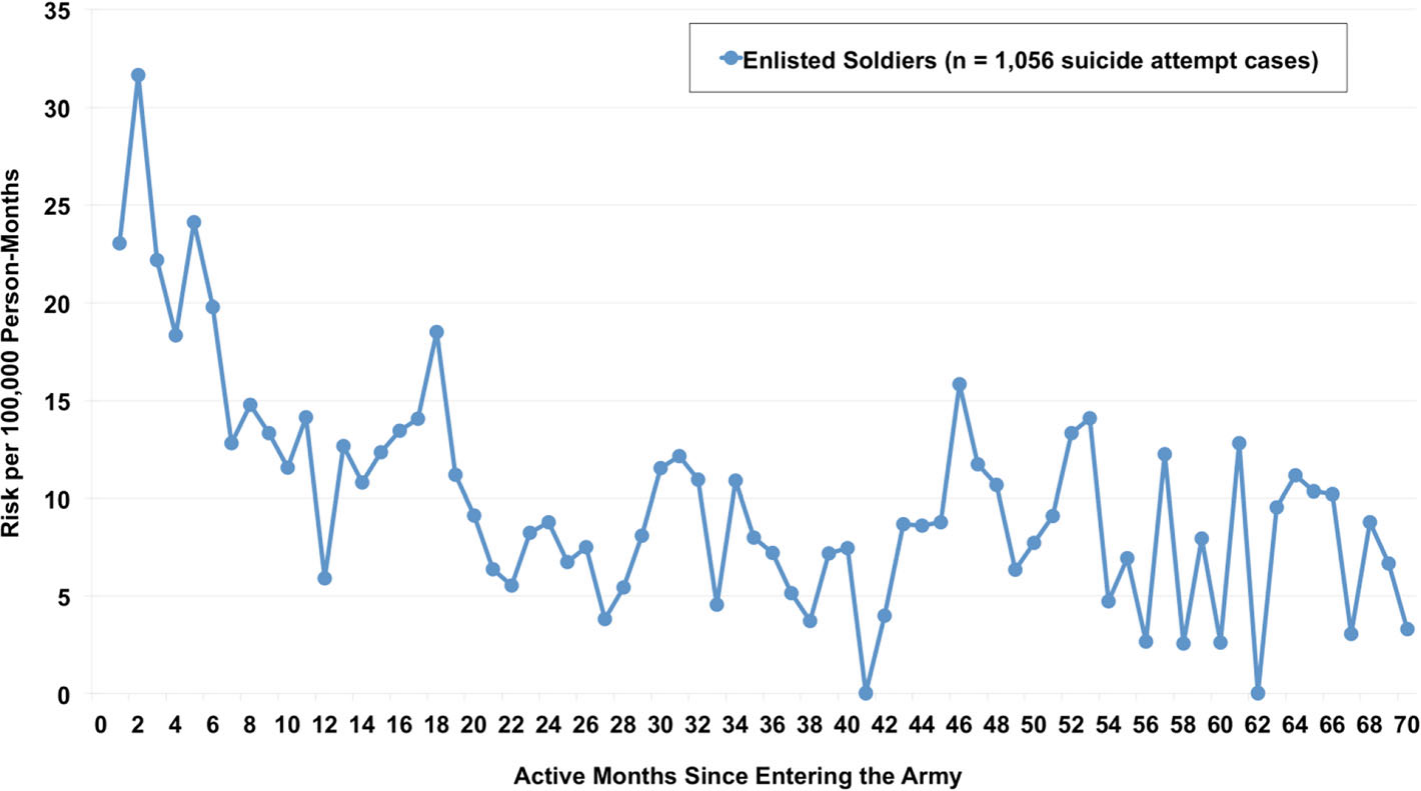
Risk of Suicide Attempt among Activated Reserve Component Enlisted Soldiers by Active Months Since Entering the Army.

A discrete-time survival model (Fig. 1) demonstrated greatly elevated risk among enlisted soldiers during their first 6 active months in the Army, with risk of suicide attempt ranging from 18 to 32/100,000 person-months and peaking in the 2nd active month of service. From the 7th through the 18th active month of service, risk generally ranged from 10 to 15/100,000 person-months, followed by a decrease to approximately 5–10/100,000 person-months through the 36th active month of service. Monthly risk was not examined among officers due to the small number of cases.

### Deployment status

We found higher odds of suicide attempt among previously deployed RC enlisted soldiers compared to those currently deployed (OR = 1.3 [95% CI: 1.1–1.6]), adjusting for socio-demographic variables (Table 3). Although the OR for never deployed was nonsignificant, a subsequent pairwise analysis found no difference between never deployed and previously deployed (χ^2^_1_ = 1.7, *p* = 0.20). Standardized risk was highest for enlisted RC soldiers who were previously deployed (SRE = 129/100,000 person-years) and lowest for those currently deployed (SRE = 93/100,000 person-years). Deployment status was not associated with suicide attempt among RC officers (Table 4).

### Mental health diagnosis

Among those who attempted suicide, 45.1% of RC enlisted soldiers and 63.8% of officers had a previous mental health diagnosis. Among attempters with a history of mental health diagnosis, 69.3% of enlisted soldiers and 56.7% of officers most recently had a diagnosis recorded in the month prior to attempting suicide. Adjusting for socio-demographics, enlisted soldiers with a mental health diagnosis in the previous month had the highest odds of attempt compared to those without a diagnosis (OR = 28.4 [95% CI = 24.7–32.7]), with odds decreasing monotonically as time since most recent diagnosis increased from 2 to 3 months (OR = 6.0 [95% CI = 4.6–7.8]) to 13 months or more (OR = 1.5 [95% CI = 1.0–2.1]) (Table 3). Pairwise analyses found that the differences between those levels were significant (ranging from χ^2^_1_ = 4.8–173.1, *p* < 0.0001–0.027). Standardized risk was highest at one month since most recent diagnosis (SRE = 1863/100,000 person-years), substantially lower at 2–3 months since diagnosis (SRE = 399/100,000 person-years), and lowest for RC enlisted soldiers with no history of diagnosis (SRE = 65/100,000 person-years). Officers with a mental health diagnosis in the previous month similarly had the highest odds of suicide attempt (OR = 49.6 [95% CI = 24.3–100.9]), with odds decreasing as time since most recent diagnosis increased from 2 to 3 months (OR = 16.4 [95% CI = 6.2–43.3]) to 4 months or more (OR = 3.1 [95% CI = 1.3–7.7]) (Table 4). Pairwise analyses found that the differences between all other levels of the variable were significant (ranging from χ^2^_1_ = 6.2–32.0, *p* < 0.0001–0.013). The standardized risk among officers decreased from 540/100,000 person-years at 1 month since diagnosis to 38/100,000 person-years at 4 or more months since diagnosis, with never-diagnosed officers having a standardized risk of 11/100,000 person-years.

## Discussion

By combining multiple administrative data systems, the current study presents the most comprehensive analysis to date of documented suicide attempts among activated RC soldiers during the wars in Iraq and Afghanistan. It complements previous RC research on suicide death [33–35] and ideation (e.g., [15–17]). The findings reveal enlisted soldiers account for the vast majority of RC suicide attempts documented during active duty, with an overall rate more than four times higher than the rate for officers. This rank-based discrepancy in risk was also observed among AC soldiers [8]. Interestingly, while the rates for RC and AC officers are comparable, the rate for RC enlisted soldiers is far lower than the published rate of 377 per 100,000 person-years for AC enlisted soldiers [8]. Previous surveys have found mixed results when comparing RC and AC soldiers on prevalence of suicide ideation and attempts [11, 12, 36]. The lower RC attempt rate in the current study may be attributable to their more limited time on active duty (when suicide attempts are captured by administrative records) relative to AC soldiers. It is also possible that RC soldiers with suicidal thoughts or other mental health symptoms that increase suicide attempt risk are less likely to be activated or remain on active duty for extended periods of time.

Among RC enlisted soldiers, the positive associations of suicide attempt with being female, younger, non-Hispanic white, less educated, and older when entering the Army are consistent with findings from the AC enlisted population, as are the higher odds among enlisted personnel who were in their first two years of service, previously deployed, and recently diagnosed with a mental health disorder [8]. Of particular note, RC and AC enlisted soldiers have a similar pattern of monthly suicide attempt risk after entering service [8], with peak risk occurring toward the end of basic training followed by a sharp decline.

Beyond potential differences in the overall attempted suicide rate among activated RC vs. AC enlisted soldiers, our findings indicate two noteworthy differences in predictor variables. First, whereas the multivariable association of marital status with suicide attempt was nonsignificant in the AC enlisted population [8], currently married RC soldiers were significantly more likely to attempt suicide than those who were never married. Interestingly, previous univariable analyses found that never married RC enlisted soldiers had higher odds of suicide attempt [9] – a finding that is consistent with the higher crude rate among never married RC soldiers in the current study – indicating that other variables in the adjusted model are influencing the association of marital status. Second, whereas being never deployed had a robust positive association with suicide attempt among AC enlisted soldiers [7, 8], it was nonsignificant among RC enlisted soldiers in adjusted models despite having the highest crude rate. The reason for this discrepancy between RC and AC soldiers is not yet known but may be attributable to differences in socio-demographic and occupational composition (e.g., the U.S. Army Reserve does not have combat units). A more detailed analysis of risk by deployment status [7] and military occupation [37] may improve understanding of how the association of deployment status with suicide attempts may differ between the RCs and AC.

Previous work found few predictors of suicide attempt among AC officers [8]. Even fewer predictors were identified in this analysis of RC officers, with female gender and recent mental health diagnosis being the only significant variables. However, with only 47 suicide attempt cases, power to detect significant associations among RC officers was limited. We plan to address this limitation in the follow-up study to Army STARRS (STARRS-LS for “longitudinal study”), which will include additional administrative data beyond the original 2004–2009 time period, allowing more officer suicide attempt cases to be captured and increased statistical power.

Several limitations in the current study are noteworthy. First, administratively recorded suicide attempts are limited to events captured by the healthcare system. These records are subject to errors in coding and clinical judgment, as well as changes in policy and procedures. Second, the data are limited to person-months during which individual RC soldiers were federally activated. Consequently, these records do not include suicide attempts or mental health diagnoses that occurred while soldiers were inactive, and time in service should not be interpreted as continuous (e.g., 12 months of active service may not correspond to 12 consecutive calendar months). Third, findings represent activated RC soldiers during 2004–2009 and may not generalize to other time periods or populations. Fourth, deployment and Army attrition are non-random events [38–40], which influences the composition of groups defined by those variables. Therefore, differences based on deployment status and active time in service should not be interpreted as within-person changes over time. For example, it is possible for soldiers who are currently deployed to have also been previously deployed. This means the currently deployed group includes soldiers on their first deployment as well as soldiers on their second deployment, third deployment, etc. Those on their first deployment are likely to have the highest risk of adverse outcomes (which could preclude subsequent deployments), whereas those on their second or third deployment are likely to be more resilient soldiers who were purposefully selected to deploy multiple times (often referred to as the “healthy warrior effect”).

## Conclusions

With the limitations in mind, the findings indicate that enlisted soldiers in their first two years of active service account for the majority of suicide attempts in the RC population, and soldiers with a recently documented mental health diagnosis are at substantially elevated risk. Although predictors are largely consistent with those found in the AC [8], there were some notable differences (e.g., marital status, deployment status). Most significantly, the intermittent nature of RC service creates unique challenges for risk assessment and intervention that are not present in the full-time AC population. AC soldiers in garrison have military healthcare access and can be closely monitored by leaders and clinicians. In contrast, deactivated members of the RCs do not have access to the military healthcare system, and they return to communities that are widely dispersed, often rural, and potentially remote, presenting obstacles to mental health screening and treatment [41]. Addressing these challenges may require new programs, such as peer-to-peer support [42], that can reach RC soldiers in their local communities.

## Additional file

Additional file 1: Supplemental Information Tables. Contains additional methodological information. (DOCX 26 kb)

## Abbreviations

AC: Active Component
CI: Confidence interval
CTS: Contingency Tracking System
DMDC: Defense Manpower Data Center
DOD: Department of Defense
DODSER: Department of Defense Suicide Event Report
HADS: Historical Administrative Data Study
ICD-9-CM: International Classification of Diseases, Ninth Revision–Clinical Modification
MDR: Military Health System Data Repository
NIH: National Institutes of Health
NIMH: National Institute of Mental Health
OR: Odds ratio
Pop %: Percentage of the population
RC: Reserve Component
SAS: Statistical Analysis System
SRE: Standardized risk estimate
STARRS: Study to Assess Risk and Resilience in Servicemembers
STARRS-LS: Study to Assess Risk and Resilience in Servicemembers-Longitudinal Study
TMDS: Theater Medical Data Store
TRAC2ES: Transportation Command Regulating And Command And Control Evacuation System
US: United States
VA: Veterans Affairs
