# Supplementary material for: Suicide attempts among activated soldiers in the U.S. Army reserve components
Source: BMC Psychiatry. 2019 Jan 18;19:31. doi: 10.1186/s12888-018-1978-2 (PMC6339319; doi:10.1186/s12888-018-1978-2)
Supplement: Supplementary file 1 — Supplemental Information Tables. Contains additional methodological information. (DOCX 26 kb) [file 12888_2018_1978_MOESM1_ESM.docx]

**Additional File 1:**

**Supplemental Information Tables**

| **Additional file 1: Table S1. Administrative data systems from the Army STARRS Historical Administrative Data Study (HADS) that were included in the current study.** | |
| --- | --- |
|  |  |
| **Database Acronym** | **Description** |
|  |  |
| DMDC/CTS | DEFENSE MANPOWER DATA CENTER (DMDC) / CONTINGENCY TRACKING SYSTEM (CTS): Collection of activation, mobilization, and deployment data. Provides information to DoD decision makers and includes a CTS Deployment File used for tracking the location of deployed personnel. |
|  |  |
| DMDC/Master Personnel & DMDC/Transaction files | DEFENSE MANPOWER DATA CENTER (DMDC) / MASTER PERSONNEL & TRANSACTION FILES: The Active Duty Master File provides an inventory of all individuals on active duty (excluding reservists on active duty for training) at a point in time. It is a standardized and centralized database of present and past members of the active duty force. Personal data elements include social security number, education level, home of record, date of birth, marital status, number of dependents, race, ethnic group, and name. Military data elements include Service, pay grade, Armed Forces Qualification Test percentile (enlisted only), source of commission (officers only), military primary duty and secondary occupation, Unit Identification Code, months of service, duty location, Estimated Termination of Service date, basic active service date, date of current rank, pay entry base date, foreign language ability, and major command code. |
|  |  |
| DODSER | DEPARTMENT OF DEFENSE SUICIDE EVENT REPORT (DODSER): Provides risk and protective factor information for suicide events. This file contains non-fatal attempts and completed suicide cases. |
|  |  |
| MDR | MILITARY HEALTH SYSTEM DATA REPOSITORY (MDR): This database contains information about medical, dental, pharmaceutical, and ancillary claims data for both in network and purchased care as well as both inpatient and outpatient treatment. Data are collected on both Army personnel and their beneficiaries. |
|  |  |
| TMDS | THEATER MEDICAL DATA STORE (TMDS): Used to track, analyze, view and manage Soldier medical treatment information recorded in the theater of operations. Features of TMDS: accessibility and visibility of service members' deployed medical records, outpatient and inpatient treatment records created in theater facilities, treatment records from other applications, reports on movement of patients, patient status and injury/illnesses. |
|  |  |
| TRAC2ES | TRANSCOM REGULATING AND COMMAND & CONTROL EVACUATION SYSTEM (TRAC2ES): A tracking system for all medical transfers across the world for all DOD services. |
|  |  |

| **Additional file 1: Table S2. International Classification of Diseases, Ninth Revision–Clinical Modification (ICD-9-CM) codes used to identify mental disorders.** | |
| --- | --- |
|  |  |
| **Included Mental Health Diagnoses** | **ICD-9-CM Codes** |
|  |  |
| Adjustment Disorder | 309, .29, .3, .4, .82, .83, .89, .9 |
|  |  |
| Dysthymic Disorder/Neurasthenia/Depression NOS | 296.82, .90, .99  300.4, .5  309.0, .1  311, .0, .1  313.1 |
|  |  |
| Major Depression | 296.2, .20, .21, .22, .23, .24, .25, .26, .3, .30, .31, .32, .33, .34, .35, .36 |
|  |  |
| Bipolar Disorder | 296.00, .01, .02, .03, .04, .05, .06, .10, .11, .12, .13, .14, .15, .16, .40, .41, .42, .43, .44, .45, .46, .50, .51, .52, .53, .54, .55, .56, .60, .61, .62, .63, .64, .65, .66, .7, .80, .81, .89  301.13 |
|  |  |
| Anxiety State/Anxiety Disorder | 300, .00, .01, .02, .09, .20, .21, .22, .23, .29, .3  309.21, .24, .28  313.0, .21, .22, .23 |
|  |  |
| Post-Traumatic Stress Disorder | 309.81 |
|  |  |
| ADHD/Learning Disorders | 314.0, .00, .01, .1, .2, .8, .9  315.00, .01, .02, .09, .1, .2, .3, .31, .32, .34, .39, .4, .5, .8, .9 |
|  |  |
| Conduct Disorder/ Oppositional Defiant Disorder | 301.7  312.4, .8, .81, .82, .89, .9  313.81  V62.83 |
|  |  |
| Eating Disorders | 307.1, .50, .51, .59 |
|  |  |
| Other Impulse Control Disorders | 312.00, .01, .02, .03, .10, .11, .12, .13, .20, .21, .22, .23, .3, .30, .31, .32, .33, .34, .35, .39 |
|  |  |
| Alcohol Induced Mental Disorders/Alcohol Dependence/Alcohol Abuse | 291.0, .1, .2, .3, .4, .5, .8, .81, .82, .89, .9  303.00, .01, .02, .03, .9, .90, .91, .92, .93  305, .0, .00, .01, .02, .03 |
|  |  |
| Drug Induced Mental Disorders | 292 |
|  |  |
| Non-Dependent Drug Abuse | 305.2, .20, .21, .22, .23, .3, .30, .31, .32, .33, .4, .40, .41, .42, .43, .5, .50, .51, .52, .53, .6, .60, .61, .62, .63, .7, .70, .71, .72, .73, .8, .80, .81, .82, .83, .9, .90, .91, .92, .93 |
|  |  |
| Drug dependence | 304 |
|  |  |
| Personality Disorders | 301.0, .1, .10, .11, .12, .20, .21, .22, .3, .4, .50, .51, .59, .6, .8, .80, .81, .82, .83, .84, .89, .9 |
|  |  |
| Non-Affective Psychosis | 295.00, .01, .02, .03, .04, .05, .10, .11, .12, .13, .14, .15, .20, .21, .22, .23, .24, .25, .30, .31, .32, .33, .34, .35, .40, .41, .42, .43, .44, .45, .50, .51, .52, .53, .54, .60, .61, .62, .63, .64, .65, .70, .71, .72, .73, .74, .75, .80, .81, .82, .83, .84, .85, .90, .91, .92, .93, .94, .95  297.0, .1, .2, .3, .8, .9  298.0, .1, .2, .3, .4, .8, .9, .90 |
|  |  |
| Somatoform/Dissociative Disorders | 300.10, .11, .12, .13, .14, .15, .16, .19, .6, .7, .80, .81, .82, .89  306.0, .1, .2, .3, .4, .50, .51, .52, .53, .59, .6, .7, .8, .9  307.54, .80, .81, .89 |
|  |  |
| Organic Mental Disorders | 290.0, .10, .11, .12, .13, .20, .21, .3, .40, .41, .42, .43, .8, .9  293.0, .1, .81, .82, .83, .84, .89, .89, .9  294.0, .1, .10, .11, .8, .9  307.20, .21, .22, .23, .3  310.0, .8, .9  317  318.0, .1, 2  319 |
|  |  |
| Sexual Disorders | 302, .0, .1, .2, .3, .4, .50, .51, .52, .53, .6, .70, .71, .72, .73, .74, .75, .76, .79, .81, .82, .83, .84, .85, .89, .9 |
|  |  |
| Sleep Disorders | 307.4, .40, .41, .42, .43, .44, .45, .46, .47, .48, .49 |
|  |  |
| Other Mental Disorders/Mental Illness | 292.85  299.00, .01, .10, .80, .81, .90, .91  300.9  307.0, .52, .53, .6, .7, .9  309.22  310.1  313.3, .82, .89, .9  316 |
|  |  |
| Traumatic Stress | 308, .0, .1, .2, .3, .4, .9 |
|  |  |
|  |  |
| **Excluded Mental Health Diagnoses** | **ICD-9-CM Codes** |
|  |  |
| Postconcussion Syndrome | 310.2 |
|  |  |
| Tobacco Use Disorder | 305.1, .10, .11, .12, .13 |
|  |  |
| Symptoms, Signs, and Ill-Defined Conditions, Mental | 797  798, .0, .1, .2, .9  799, .0, .01, .02, .1, .2, .21, .22, .23, .24, .25, .29, .3, .4, .8, .81, .82, .89, .9 |
|  |  |
| Stressors/Adversities | V40.0, .00, .1, .2, .20, .3, .30, .9, .90  V61, .0, .01, .02, .03, .04, .05, .06, .07, .08, .09, .2, .20, .21, .22, .23, .24, .29, .3, .4, .41, .42, .49, .8, .9  V62, .0, .1, .1 0, .2, .20, .21, .22, .29, .3, .4, .5, .8, 80, .81, .810, .811, .812, .82, .89, .9, .90  V69.4, .5, .9 |
|  |  |
| Marital Problems | V61.1, .10, .11, .12 |
|  |  |
| Suicidal Ideation | V62.84 |
|  |  |
| Prior History of Mental Disorders | V11.0, .1, .2, .3, .8, .80, .9, .90  V66.3  V67.3 |
|  |  |
| Indicator of Impulsivity and Risky Behavior | V69.2, .3 |
|  |  |
| Self-Damaging Behavior | V69.8 |
|  |  |
